# 

**Published:** 1900-03

**Authors:** 


### CONTENTS.

ORIGINAL COMMUNICATIONS—

MORPHINISM. By. C. C. Stockard, M.D., Atlanta, Ga.......... [illegible]
NOSE AND THROAT AFFECTIONS. By C. H. Powell, A.M., M.D., St. Louis, Mo.......... [illegible]
A MEDICO-RELIGIOUS CHARITY—THE GUILD OF MERCY. By W. Thornton Parker, M.D., Westboro, Mass.
THERAPEUTIC APPLICATION OF BLISTERS. By C. D. Hurt, M.D., Atlanta, Ga..........
DIAGNOSIS OF DISEASES OF THE STOMACH. By Charles D. Aaron, M.D., Detroit, Mich..........
LAW AND MEDICINE. By C. A. F. Lindorme, Ph.D., M.D., Chicago, Ill..........

CORRESPONDENCE—

UNITED CONFEDERATE VETERANS' REUNION AT LOUISVILLE..........

EDITORIAL NOTES AND COMMENTS—

QUACKERY, AND A SUGGESTION..........
PARTURITION AMONG THE ESQUIMAUX..........
HOMING PIGEONS AND THE COUNTRY DOCTOR..........

NEWS AND NOTES..........
CONTENTS CONTINUED..........

